# Significant Enhancement of the Visible Light Photocatalytic Properties in 3D BiFeO_3_/Graphene Composites

**DOI:** 10.3390/nano9010065

**Published:** 2019-01-05

**Authors:** Jiquan Li, Youyan Wang, Huan Ling, Ye Qiu, Jia Lou, Xu Hou, Sankar Parsad Bag, Jie Wang, Huaping Wu, Guozhong Chai

**Affiliations:** 1 Key Laboratory of E&M, Zhejiang University of Technology, Ministry of Education & Zhejiang Province, Hangzhou 310014, China; lijq@zjut.edu.cn (J.L.); m15958026031@163.com (Y.W.); ldorothyh07@163.com (H.L.); qiuye2024@163.com (Y.Q.); 2 Piezoelectric Device Laboratory, Department of Mechanics and Engineering Science, Ningbo University, Ningbo 315211, China; jiajia_smile@163.com; 3 Department of Engineering Mechanics, School of Aeronautics and Astronautics, Zhejiang University, Hangzhou 310027, China; emhouxu@zju.edu.cn (X.H.); sankar.bag19@gmail.com (S.P.B.); jw@zju.edu.cn (J.W.)

**Keywords:** BiFeO_3_, hydrothermal, photocatalysis, methylene blue

## Abstract

Bismuth ferrite (BiFeO_3_, BFO) submicron cubes and 3D BFO/graphene composite materials were synthesized by a simple hydrothermal process. The crystallization processes of the 3D BFO/graphene composites with different graphene oxide (GO) concentrations were studied for their visible light photocatalytic properties. Compared to the single BFO submicron cubes, 3D BFO/graphene composites have greatly improved photocatalytic activity. A high photocatalytic performance is obtained at a GO concentration of 3 mg/mL, with the degradation rate of methylene blue (MB) dye reaching up to 92% in 140 min. The enhancement of photocatalytic activity can be attributed to the large specific surface area and 3D architecture of 3D composites, which provide more transport paths to effectively improve the separation rate of photo-generated electrons and holes. Therefore, 3D BFO/graphene composites have a broad prospect of application in the field of photocatalysis.

## 1. Introduction

In the past decades, as a kind of green and safe purification technology, photocatalysis has been widely applied in the fields of solar energy conversion and environmental purification, and it has attracted the attention of many scholars [1,2,3,4,5,6,7]. In the photocatalytic reaction, traditional semiconductor materials such as CdS [8,9], ZnO [10,11], and TiO_2_ [12,13,14,15,16,17] are widely studied as photocatalysts. However, these semiconductor materials have low photocatalytic activity under visible light irradiation due to their wide band gaps. In recent years, ferroelectric materials have been given great attention in various fields [18,19,20,21,22]. BiFeO_3_ (BFO) as a ferroelectric material has become a hot material in the field of photocatalysis because of its narrow band gap [23,24,25,26,27] and photovoltaic effect, which extend the light response range to the visible region and greatly improve the utilization rate of solar energy [28,29]. For example, Gao et al. revealed that BFO displayed high photoactivity for the degradation of methyl orange under exposure to visible light [30]. Efforts have been made for the improvement of BFO’s photocatalytic performance, by elemental doping of BFO for band engineering [31], as well as by preparing BFO samples with large specific surface areas, i.e., nanoparticles [32], nanowires [33,34], micro cubes [35], and porous thin films [36]. In addition, the Basu Research Group applied rGO/BFO composite materials for the photocatalytic degradation of RhB dye (94%) with a degradation rate constant of 1.86 × 10^−2^ min^−1^, which is 3.8 times faster than that of pure BFO, demonstrating that graphene can help to improve the photocatalytic activity of BFO material [37]. 

Carbon materials have many excellent physical and chemical properties, and are widely used in many fields [38,39,40,41,42]. The advantages of graphene-based photocatalysts lie in the unique properties of graphene, such as high electron conductivity and excellent charge mobility, which lead to longer lives of photo-generated electrons and holes pairs produced by semiconductor nanoparticles on graphene sheets, and thus higher photocatalytic efficiency [43,44,45]. However, the high specific surface area of monodisperse graphene is decreased by the aggregation of two-dimensional graphene due to the strong attraction between the layers. In order to avoid this problem, many works have focused on the 3D graphene composite [46,47,48,49,50]. The 3D graphene composite has a three-dimensional porous network structure (combined with hydrogen bonds through the interaction between α and β), which has a larger surface area, higher mechanical strength, and faster proton and electron transmission speed than the two-dimensional structure [51,52,53], and also good adsorption capacity [54]. Chen et al. [55] successfully synthesized 3D graphene aerogel-supported Ag and Ag/Ag_3_PO_4_ heterostructures to degrade different dyes with high catalytic activities in the visible light range. Zheng et al. [37] synthesized 3D graphene aerogel (g–C_3_N_4_–TiO_2_–GA) composites by combining the hydrothermal method with freeze-drying. The adsorptivity for RhB was as high as 96.5% in the dark, and the overall removal efficiency reached up to 98.4% under visible light irradiation within 60 min.

Nevertheless, little work has been done to systematically study the effects of graphene addition on the morphologies and photocatalytic properties of 3D BFO/graphene composites, and the corresponding mechanism is not clear [56], which motivates us to do further study. In this paper, the 3D BFO/graphene composites are prepared by the hydrothermal method. The pure phase and regular configuration of BFO submicron cubes are formed by adjusting the hydrothermal time and the pH of precursors during the hydrothermal process. We analyzed the effects of different concentrations of GO solution on the photocatalytic properties of 3D BFO/graphene composites.

## 2. Materials and Methods

### 2.1. Synthesis of BFO and 3D BFO/Graphene Composites

BFO submicron cubes were synthesized by a simple hydrothermal method [57,58], and all the chemicals were purchased from Aladdin. Bi(NO_3_)_3_•5H_2_O and Fe(NO_3_)_3_•9H_2_O crystals were weighed in a molar ratio of 1:1, and then dissolved in 50 mL of deionized water by magnetic stirring. NaOH solution (2 M) was slowly added into the mixed solution containing Bi^3+^ and Fe^3+^ under magnetic stirring, producing a large amount of orange precipitate. Then, 2 mL of hydrogen peroxide (30%) was added into the reactor, and the solution was placed in an environment of 200 °C for 72 h. After the hydrothermal reaction, the reactants were centrifuged 4–5 times at a speed of 8000 rpm for 10 min each time. Finally, BFO submicron cube powder was obtained by drying the reactants.

GO was synthesized from natural graphite powder by a modified Hummer’s method, as mentioned in previous works [59,60,61]. BFO submicron cubic powder (1 g) was added into GO solution (40 mL) with different concentrations. Ascorbic acid was added to the mixture of GO and BFO as the reducing agent, such that the mass ratio of GO to ascorbic acid was 1:2. After uniform mixture by magnetic stirring, the solution was transferred to a reaction vessel for the hydrothermal reaction. The hydrothermal time was 6 h, and the hydrothermal temperature was 160 °C. In this process, GO was reduced to rGO. After the hydrothermal reaction, the reactants were put into a freeze-dryer for drying.

### 2.2. Photocatalytic Experiments

In the photodegradation experiments, the CHI660E electrochemical workstation manufactured by Shanghai Chenhua Company was used for electrochemical performance tests. The test mode adopted a three-electrode system. The BFO/graphene composite-coated ITO-glass substrate, Pt wire, and Ag/AgCl electrode were used as the working electrode, counter electrode, and reference electrode, respectively. The light source in the photocatalysis experiment was a xenon lamp with a power of 150 W, and the parallel light power density of the xenon lamp was 70 mW cm^−2^ (which was tested by the actinometer TASI TA8120, China). MB was chosen as the degradation material to examine the photodegradation of BFO and 3D BFO/graphene composites [62]. Before photocatalytic tests, the samples with different concentrations of GO were horizontally immersed into 20 mL MB solution (40 mg/L) for three hours to achieve the adsorption–desorption equilibrium, and then continually irradiated under visible light. During this process, 5 mL of MB solution was directly collected every hour to calculate the concentration of residual MB. The absorption intensity at its maximum absorbance wavelength was measured.

### 2.3. Characterization

The morphologies of the BFO and BFO/graphene composites were observed using scanning electron microscopy (SEM, S-4800, Hitachi, Japan 800). The Raman analysis was performed on a Raman spectroscope (Renishawin Via Raman Microscope) with an argon-ion laser at an excitation wavelength of 514 nm. The crystal structures of the BFO and 3D BFO/graphene composites were characterized by X-ray diffraction (XRD, Siemens D5005, Siemens, Germany). The surface areas of the BFO and BFO/graphene composites were estimated by measuring the nitrogen adsorption–desorption isotherms on a Micromeritics ASAP 2020 M micropore analysis system at 77 K. A spectrometer (UV–vis, UV-2401PC spectrometer, Shimadzu, Japan) was used to analyze the optical reflection of the samples and the maximum absorbance wavelength of residual MB dye after photodegradation experiments. The thermogravimetric analyses (TGA) of BFO and 3D BFO/graphene composites (B-3D3) were performed by a thermogravimetric analyzer (PYRIS 1, PerkinElmer, USA) in an air atmosphere of 25–800 °C. The Total Organic Carbon (TOC) experiment was carried out by using a total organic carbon analyzer (Liquid Toc2, Elemenear, Germany).

## 3. Results and Discussion

### 3.1. Effects of Hydrothermal Time and pH of Precursor on the Crystallization of BFO

**Figure 1:**
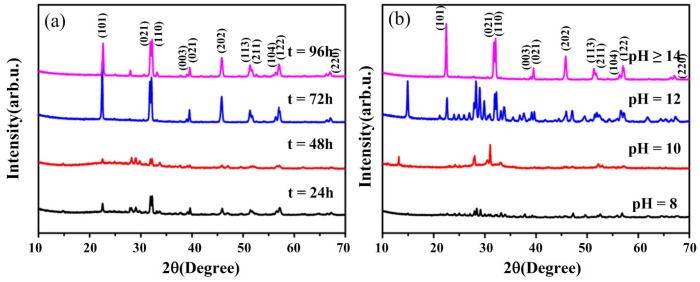
X-ray diffraction patterns of different (**a**) hydrothermal times and (**b**) precursor pH values.

The quality of BFO at different hydrothermal times and pH values of the precursor can be readily observed from the XRD patterns with the same hydrothermal temperature (200 °C). In Figure 1a, we can see that BFO cannot be normally crystallized when the hydrothermal time is 24 or 48 h. However, BFO can be crystallized well when the hydrothermal time is more than or equal to 72 h. The characteristic peak located at 2*θ* of 33.11° indicates that BFO has the perovskite-type rhombohedra structure [31]. However, there is a hybrid phase when the hydrothermal time is more than 72 h and the product contains common impurities such as Bi_2_Fe_4_O_9_ and Bi_2_O_3_/Fe_2_O_3_. As shown in Figure 1b, BFO cannot be crystallized when the pH value of the precursor is 8 or 10. When the pH value of the precursor is 12, BFO can be crystallized well, but there are a large number of heterogeneous phases. BFO can be crystallized well and the pure phase is obtained when the pH value of the precursor is more than 14, as seen in Figure 1b. In other words, the precursor concentration should be controlled in a strong alkaline environment. Thus, we conclude that the optimum conditions to form the pure phase of BFO are a hydrothermal time of 72 h and the precursor pH value being more than 14.

**Figure 2:**
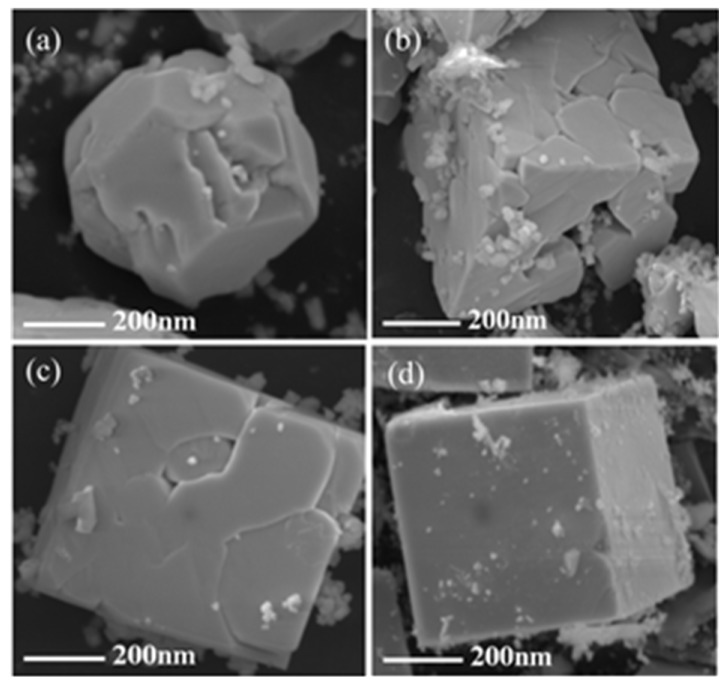
SEM images of BFO nanoparticles with different precursor pH values: (**a**) pH = 8; (**b**) pH = 10; (**c**) pH = 12; (**d**) pH ≥ 14.

In addition, the pH of the precursor affects the morphology of BFO. Figure 2a displays that the BFO nanoparticles are agglomerating into nuclei, and are not in the square phase yet (pH = 8). In Figure 2a,b, the BFO submicron cube is gradually formed, but relatively loose with a rough surface (pH = 10). As the BFO nanoparticles continue to agglomerate, the cube is already compact, and its surface tends to be smooth in Figure 2c (pH = 12). It is worth noting that a BFO submicron cube with a smooth surface and stable shape is eventually formed with pH = 14 (Figure 2d). It is proven that the morphology of BFO nanoparticles can be adjusted by the pH of the precursor.

### 3.2. Effects of Graphene Oxide Concentration on the Crystallization of BFO

**Figure 3:**
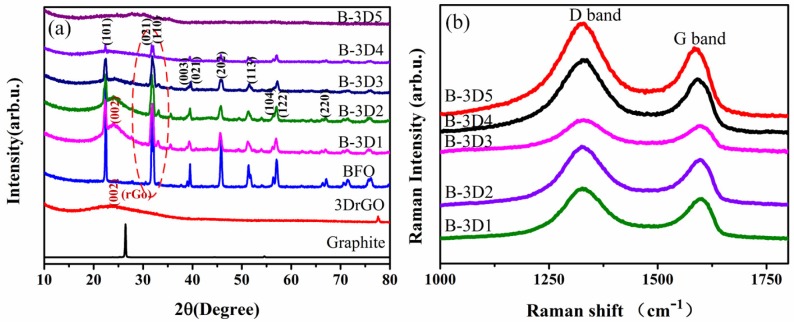
(**a**) X-ray diffraction patterns and (**b**) Raman spectra of the 3D BFO/graphene composite samples with different GO concentrations.

It has been demonstrated that the single BFO submicron cube without the addition of GO has pure perovskite structure with good crystallinity. The red circle part in Figure 3a shows that the crystallinity of BFO decreases gradually with the increase of GO concentration. From Figure 3a, we can also see that the broad peak at 24.8 degrees is related to the characteristic peak of stacked multilayer graphene [59,63]. In addition, the Raman data shown in Figure 3b can also prove that we prepared the graphene material. Raman graphs of 3D BFO/graphene composites with different GO concentrations are presented in Figure 3b. The D peak represents the circular breathing mode of the sp^2^ hybrid carbon atom ring in graphene and the defects and disorder of the carbon lattice [39]. In high quality graphene, D peaks are generally very weak. When the GO solution concentration is 3 mg/mL, the D peak of the synthesized 3D BFO/graphene composite is weaker than other concentrations, indicating that the defects in the composite of this concentration are less.

As mentioned above, the formation of pure phase BFO submicron cubes requires a strongly alkaline environment. The addition of GO destroys the alkaline growth environment of the BFO submicron cube, so when the concentration of GO is more than 3 mg/mL, the crystallinity of the BFO submicron cube is not good. However, when the concentration of GO is less, the BFO submicron cube tends to generate miscellaneous items. To sum up, the optimized concentration of GO is 3 mg/mL. 

**Figure 4:**
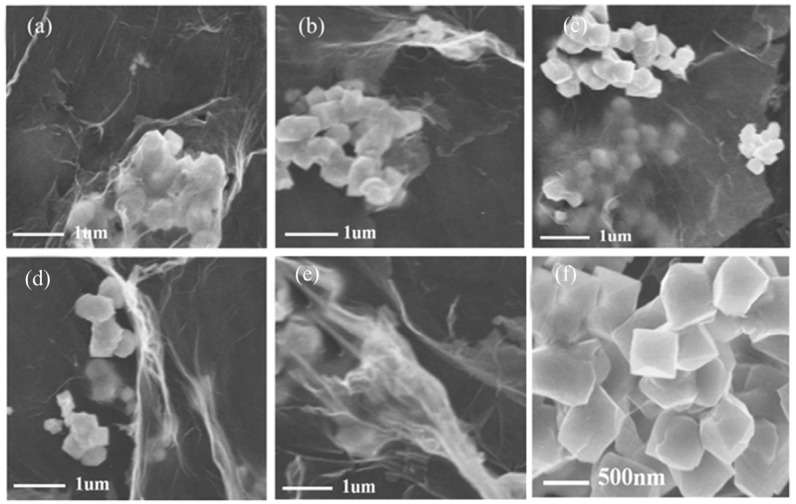
TEM images of 3D BFO/graphene composites: (**a**) B-3D1; (**b**) B-3D2; (**c**) B-3D3; (**d**) B-3D4; (**e**) B-3D5; (**f**) B-3D3.

Furthermore, Figure 4 shows the TEM images of 3D BFO/graphene composites with different concentrations of GO. When the concentration of GO is 1 or 2 mg/mL, the microcrystals of BFO are agglomerated, and the original submicron cubic shape is irregular, as shown in Figure 4a,b. The low concentration of GO makes BFO submicron cubes produce miscellaneous terms, as shown in Figure 3a.When increasing the concentration of GO from 1 to 3 mg/mL, more regular morphologies and uniform distributions of BFO submicron cubes are obtained, as shown in Figure 4c,f. Meanwhile, the morphologies of the BFO submicron cubes have been destroyed and the crystallization degree is not high under the concentrations of 4 and 5 mg/mL, as shown in Figure 4d,e. The surface areas of BFO/graphene composites were estimated by measuring the nitrogen adsorption–desorption isotherms. The surface area increases from 21.3 m^2^/g for the BFO sample to 55.3 m^2^/g for BFO/graphene composites (3 mg/mL), indicating the porous structure of the composites.

### 3.3. Photocatalytic Performance

**Figure 5:**
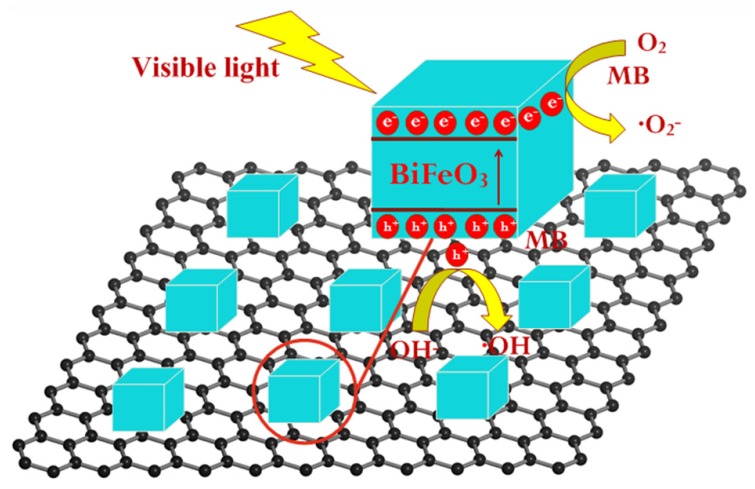
The photocatalytic mechanism diagram of 3D BFO/graphene composites.

First, we studied the photocatalytic mechanism of BFO/graphene composites under visible light. A photocatalytic mechanism diagram is shown in Figure 5. BFO is stimulated by visible light to produce electrons and holes. In this process, 3D graphene increases the number of active sites on its surface due to the large surface area [64]. As a transport carrier, 3D graphene can effectively separate electrons and holes, enabling them to participate in subsequent redox reactions, generate active free radicals, and enhance photocatalytic activity. Moreover, 3D BFO/graphene composites have narrow band gaps, which can utilize visible light more effectively and improve the photocatalytic performance.

**Figure 6:**
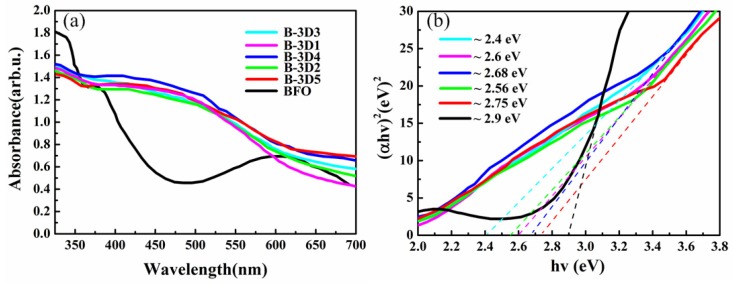
UV—vis absorption spectra (**a**) and corresponding (*αhv*)^2—^*hv* curves (**b**) of 3D BFO/graphene composites.

Therefore, we tested the optical absorption and obtained the band gap values of the 3D BFO/graphene composites under visible light. Figure 6a presents the ultraviolet-visible absorption spectrum of 3D BFO/graphene composites with different GO concentrations. Six samples could absorb light energy in both the ultraviolet and visible regions. It should be pointed out that BFO had strong absorption peaks at about 430 nm. When BFO combined with 3D graphene, there was strong absorption across the entire wavelength range, and the addition of graphene affected the absorption of BFO submicron cubes at 350–600 nm. The (*αhv*)^2^—*hv* curve shown in Figure 6b could be calculated from Figure 6a. The band gap values of BFO and BFO/graphene composites could be estimated based on Equation (1) [65]:

(1) $${\left(\alpha h\nu \right)}^{2}=A\left(h\nu -Eg\right),$$

where *α* (L/g·cm^−1^) is the absorption coefficient, *h* (eV·s) is the Planck constant, *v* (m^−^^1^) is the ratio of the speed of light with the wavelength, the *hv* value is 1240/wavelength, *Eg* (eV) is the band gap, and A is a constant.

The band gap could be estimated from the (*αhv*)^2^ versus *hv* plot by extrapolating the linear portion of (*αhv*)^2^ to the energy (*hv*) axis at *α* = 0 [66]. Table 1 shows the band gap values of the 3D BFO/graphene composites with different GO concentrations. We found that the band gap can be reduced by the addition of GO. When the concentration of GO is 3 mg/mL, the band gap is at its minimum with a value of 2.4 eV. The introduction of graphene makes the band gap of 3D BFO/graphene composites smaller than BFO because the interaction between graphene and BFO affects the band gap of the composite material. Since the light response range of the 3D BFO/graphene composites is in the visible region, the composite material has potential as a catalyst.

**Figure 7:**
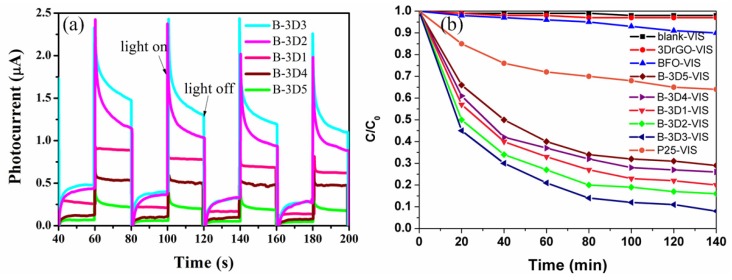
(**a**) P—T curves for the 3D BFO/graphene composites and (**b**) the degradation rate of MB dyes from different materials under visible light irradiation.

We further tested the photocurrent change (P—T curves) of 3D BFO/graphene composites with different GO concentrations using a three-electrode system. The BFO/graphene composite-coated ITO conductive glass was used as the working electrode. The lighting condition was designed by adjusting the light switch with a time interval of 20 s. When lighting was added, the working electrode absorbed the light and produced a large number of electrons and holes. The electrons were exported through the ITO conductive glass, and the holes were trapped by the electrolyte to generate a current. In the absence of light, the light current was zero. Repeated cycles and good repeatability indicated that the physical and chemical stability of the photoanode prepared in this work is excellent. As it can be seen from Figure 7a, the photocurrent intensity of the 3D BFO/graphene composite is strongest when the concentration of GO is 3 mg/mL (B-3D3), indicating that the separation rate of the electrons and holes is highest in this composite.

Finally, we tested the degradation of MB dye by using the 3D BFO/graphene composites with different GO concentrations. We had five copies of each sample and did five experiments, and finally calculated the average value. The amount of catalyst was 0.05 g, and the concentration of MB solution (20 mL) was 40 mg/L. Before the photocatalytic tests, the 3D BFO/graphene composites were added to the solutions of MB dye for three hours to achieve the adsorption–desorption equilibrium. Since the characteristic absorption did not obviously change before and after the adsorption–desorption equilibrium, we suggest that the adsorption of MB dye on the BFO/graphene composites was negligible. The specific reaction process is as follows:

$$\begin{array}{c} {\mathrm{BiFeO}}_{3}+\;\mathrm{hv}\to h^{+}+{\;e}^{-}\\ h^{+}+{\;e}^{-}\to \mathrm{Energy}\\ {2H}_{2}O\;+{\;4h}^{+}\to O_{2}+{\;4H}^{+}\\ e^{-}+{\;O}_{2}\to \cdot O_{2}{}^{-}\\ O_{2}+{\;H}^{+}\to {\mathrm{HO}}_{2}\cdot \\ {2\mathrm{HO}}_{2}\cdot \to O_{2}+{\;H}_{2}O_{2}\\ {2O}_{2}{}^{-}+{\;2H}^{+}\to H_{2}O_{2}+{\;O}_{2}\\ O_{2}{}^{-}+{\;\mathrm{HO}}_{2}\cdot \to H_{2}O_{2}+{\;O}_{2}\\ h^{+}+{\;H}_{2}O\to \cdot \mathrm{OH}\;+{\;H}^{+}\\ \cdot \mathrm{OH}\;+\;\mathrm{MB}\to \cdots \to {\mathrm{CO}}_{2}+{\;H}_{2}O\\ h^{+}+\;\mathrm{MB}\to \cdots \to {\mathrm{CO}}_{2}+{\;H}_{2}O \end{array}$$

Here, e^−^ and h^+^ represent electrons and holes, respectively, which react with oxygen dissolved in water to form O_2_^−^ and highly reactive radicals (⋅OH and ⋅OOH).

**Figure 8:**
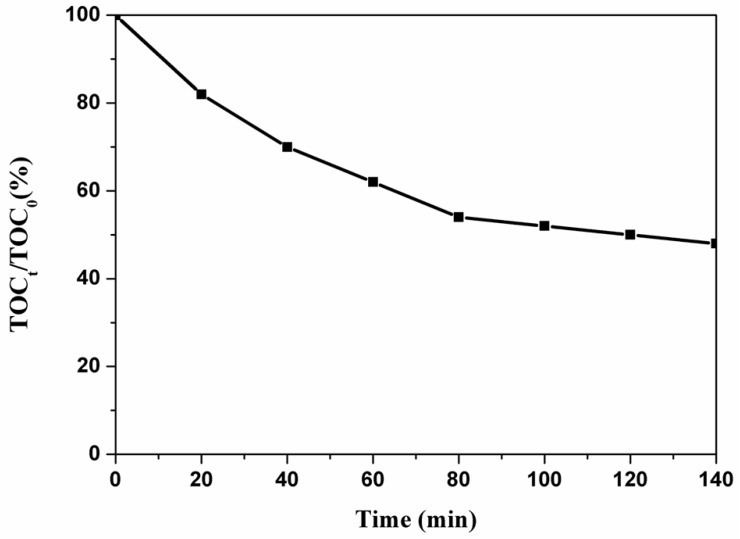
Total Organic Carbon (TOC) content of the degrading solution using the B-3D3 sample.

Figure 7b shows the degradation curve of MB dye by 3D BFO/graphene composites with different GO concentrations. It is obvious that 3D graphene does not have the ability to degrade MB solutions within 140 min. The single BFO submicron cube has weak degradation effects as shown by the blue line in Figure 7b. The degradation ability of 3D BFO/graphene composites is greatly improved compared to the single BFO and P25 materials. The reason is that the 3D BFO/graphene composites have large specific surface areas and electron transport capacities. The three-dimensional structures of the composites provide more paths for electron transport, which helps to separate electrons and holes, reduces the recombination rate of electrons and holes, and improves the rate and effect of subsequent catalytic reactions. In conclusion, the 3D BFO/graphene composites prepared with different GO concentrations have different degradation capacities for MB dye. When the GO concentration was 3 mg/mL (B-3D3-VIS), the degradation rates of MB were 90%, 92%, 93.5%, 91.5%, and 93%, respectively, in five parallel experiments. We used their average value (92%) as the experimental result, and the experimental error was less than 2%, which indicates good stability. We further tested the proportion of rGO in the composites (B-3D3) (Figure S1), which was approximately 8.0%, indicating that most of the GO was restored to rGO. Additionally, we also examined the organic carbon content of the degradation solution. After the dark reaction, the TOC of the solution was 25.58 mg/L. When the photocatalytic reaction time was 140 min, the TOC of the solution was 12.28 mg/L, demonstrating a high degradation rate of about 52% for organic carbon, as shown in Figure 8.

## 4. Conclusions

In this paper, we prepared BFO submicron cubes by the hydrothermal method. It was found that the optimum conditions to form pure phase BFO submicron cubes with regular morphology are a hydrothermal time of 72 h and a precursor pH value of more than 14. Different 3D BFO/graphene composites were fabricated by adjusting the GO concentration. The results show that adding 3D graphene can adjust the band gaps of 3D BFO/graphene composites, thus greatly improving the photocatalytic activity of the composites. Moreover, better structure and photocatalytic performance of the 3D BFO/graphene composites can be obtained when the GO concentration is 3 mg/mL. Under visible light irradiation, the degradation rate of MB dye using these 3D BFO/graphene composites reaches 92% in 140 min. The enhancement of photocatalytic activity in visible light can be attributed to the large specific surface areas and three-dimensional architecture of composites, which can provide more transport paths and effectively improve the separation rate of photo-generated electrons and holes.

## Figures and Tables

**Table 1:** The band gaps of 3D BFO/graphene composites.

Sample	BFO	B-3D1	B-3D2	B-3D3	B-3D4	B-3D5
**Eg (eV)**	2.90	2.60	2.56	2.40	2.68	2.75

## Supplementary Materials

The following are available online at http://www.mdpi.com/2079-4991/9/1/65/s1, Figure S1: TGA of BFO and 3D BFO/graphene composite.
