# Supplementary material for: Significant Enhancement of the Visible Light Photocatalytic Properties in 3D BiFeO3/Graphene Composites
Source: Nanomaterials (Basel). 2019 Jan 5;9(1):65. doi: 10.3390/nano9010065 (PMC6359105; doi:10.3390/nano9010065)
Supplement: Supplementary file 1 [file nanomaterials-09-00065-s001.pdf]

Supporting Information Significant Enhancement of the Visible Light Photocatalytic Properties in 3D $BiFeO_3$/Graphene Composites

Jiquan Li [1], Youyan Wang [1], Huan Ling [1], Ye Qiu [1], Jia Lou [2], Xu Hou[3], Sankar Parsad Bag[3], Jie Wang [3], Huaping Wu [1,*] and Guozhong Chai [1,*]

[1] Key Laboratory of E&M, Zhejiang University of Technology, Ministry of Education & Zhejiang Province, Hangzhou 310014, China; lijq@zjut.edu.cn (J.L.); m15958026031@163.com (Y.W.); ldorothyh07@163.com (H.L.); qiuye2024@163.com (Y.Q.);

[2] Piezoelectric Device Laboratory, Department of Mechanics and Engineering Science, Ningbo University, Ningbo, Zhejiang 315211, China; jiajia_smile@163.com (J.L.)

[3] Department of Engineering Mechanics, School of Aeronautics and Astronautics, Zhejiang University, Hangzhou 310027, China; emhouxu@zju.edu.cn; sankar.bag19@gmail.com; jw@zju.edu.cn (J.W.)

* Correspondence: wuhuaping@gmail.com, chaigz@zjut.edu.cn; Tel.: +86-15158867224 (H.W.)

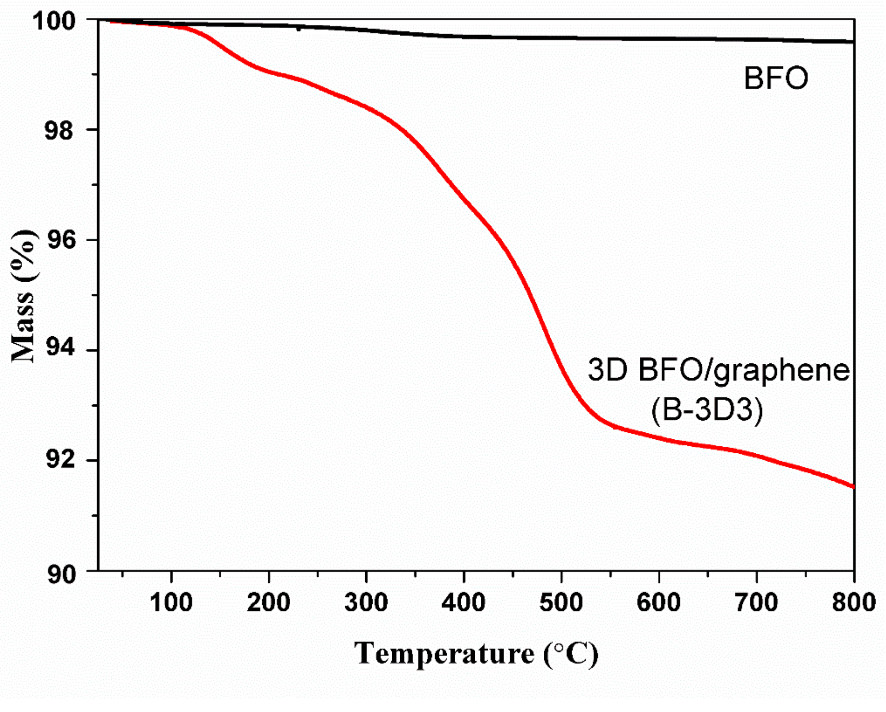

Figure S1. TGA of BFO and 3D BFO/graphene composite.

As shown in Figure S1, there is a 0.5% weight loss in the BFO sample, which may be caused by moisture or organic impurities in the sample. The greater weight loss in the BFO/graphene composite is due to the degradation of rGO. Therefore, we speculate that the mass fraction of rGO in the composite is approximately 8.0%.
